# An Early Morning Sputum Sample Is Necessary for the Diagnosis of Pulmonary Tuberculosis, Even with More Sensitive Techniques: A Prospective Cohort Study among Adolescent TB-Suspects in Uganda

**DOI:** 10.1155/2012/970203

**Published:** 2012-12-04

**Authors:** Willy Ssengooba, David P. Kateete, Anne Wajja, Eric Bugumirwa, Gerald Mboowa, Carolyn Namaganda, Germine Nakayita, Maria Nassolo, Francis Mumbowa, Benon B. Asiimwe, James Waako, Suzanne Verver, Philippa Musoke, Harriet Mayanja-Kizza, Moses L. Joloba

**Affiliations:** ^1^Department of Medical Microbiology, School of Biomedical Sciences, Makerere University College of Health Sciences, Kampala, Uganda; ^2^Infectious Diseases Institute, Makerere University College of Health Sciences, Kampala, Uganda; ^3^Iganga/Mayuge Demographic Surveillance Sites, School of Public Health, Makerere University College of Health Sciences, Kampala, Uganda; ^4^KNCV Tuberculosis Foundation, The Hague and CINIMA, Academic Medical Centre, Parkstraat 17, 2514 JD The Hague, The Netherlands

## Abstract

The World Health Organization (WHO) recommends collection of two sputum samples for tuberculosis (TB) diagnosis, with at least one being an early morning (EM) using smear microscopy. It remains unclear whether this is necessary even when sputum culture is employed. Here, we determined the diagnostic yield from spot and the incremental yield from the EM sputum sample cultures among TB-suspected adolescents from rural Uganda. Sputum samples (both spot and early-morning) from 1862 adolescents were cultured by the Lowenstein-Jensen (LJ) and Mycobacterium Growth Indicator Tube (MGIT) methods. For spot samples, the diagnostic yields for TB were 19.0% and 57.1% with LJ and MGIT, respectively, whereas the incremental yields (not totals) of the early-morning sample were 9.5% and 42.9% (*P* < 0.001) with LJ and MGIT, respectively. Among TB-suspected adolescents in rural Uganda, the EM sputum culture has a high incremental diagnostic yield. Therefore, EM sputum in addition to spot sample culture is necessary for improved TB case detection.

## 1. Background

Tuberculosis (TB) remains a global emergency, causing high mortality and morbidity particularly in sub-Saharan Africa [1]. Some studies have shown that the incidence of TB in adolescents (12–18 years old) has increased by 22% compared with a 38% decrease in children less than 5 years old [2]. 

Although there is scant data on TB in adolescents in Uganda and worldwide in general, their protective response against *Mycobacterium tuberculosis* (MTB) infection seems to be less effective [3]. Adolescents also have unique clinical presentations for TB; they are more asymptomatic and are more likely to have cavitary disease [4]. Reports indicate that many adolescents with active TB are diagnosed during late stage of the disease [2]. Additionally, the demographic and clinical characteristics of adolescents with TB differ from adults and children [5]. Furthermore, it is quite difficult to obtain quality sputum samples, which makes TB diagnosis among adolescents challenging. Indeed, many adolescents with TB are prone to producing smear negative sputum samples [6]. 

The World Health organization (WHO) and the International Union Against Tuberculosis and Lung Diseases (IUATLD) recommended collection of two sputum samples for smear microscopy with at least one being an early-morning (EM) sample. This aimed at reducing the workload per serial sample examined [7, 8]. However, these recommendations seem to work in settings serving the general population where external quality assurance (EQA) methods are well established. As such, those recommendations may not address the number of samples required in other situations where sputum culture is indicated. 

The definitive diagnosis of TB depends mainly on culture of MTB from the clinical samples [9]. According to the mathematical models by Dowdy et al., expanded use of TB culture may have an immediate impact on TB burden in that it can reduce TB rates in high burdened countries [10]. However, this concept remains less applied in resource limited countries that are most burdened by TB. This is largely due to the high expenses and cost of infrastructure required [11]. For instance, there is only one public TB culture facility in Uganda, located in Kampala, the capital.

Despite the move to expand and improve laboratory capacity to perform TB culture [12], and the availability of automated liquid culture systems which requires less labor [13], little has been done to assess the impact and cost effectiveness including operational studies for performing TB culture in resource limited countries. The situation is even worse when adolescents are involved in that culture would be the most appropriate for diagnosis [14]. 

Whereas the number and type of sputum samples required for smear microscopy have been standardized, the number and type of sputum samples required for culture have not been well standardized. Furthermore, while there is a significant diagnostic gain from EM sputum samples when using smear microscopy, it is not clear whether this gain remains significant when using more sensitive techniques such as culture particularly among adolescents. This study therefore aimed at determining the diagnostic yield of the spot and the incremental yield of EM sputum sample cultures for diagnosis of TB among adolescents in rural Uganda.

## 2. Methods 

### 2.1. Study Design and Setting

This study was a subcomponent of a large observational study undertaken by Makerere University School of Public Health, which enrolled 5000 adolescents in rural Uganda, in preparation for TB vaccine trials. The study was done in a Demographic Surveillance Site (DSS) located in Iganga and Mayuge districts in Eastern Uganda (~150 km from Kampala, the capital).

The adolescent TB cohort (12–18 years of age) enrolled 5,000 participants from both primary and secondary schools within the DSS. It enrolled participants with a positive Tuberculin Skin Test (TST) and at least one TB related symptom or history of contact with a TB diseased person. Participants who had plans of relocating within the study period or those medically unfit for collection of sputum were excluded. Clinical evaluation for TB disease included giving two sputum samples; one collected on spot under supervision (spot sample) on day one and another on day 2 by the participant without supervision (EM sample).

Sputum samples were obtained daily from the participants by nurses and transported in cold boxes (temperature ≤ 8°C) to a reference mycobacteriology laboratory (biosafety level 3) where culture and analysis were done. This laboratory is located in the Department of Medical Microbiology, Makerere University College of Health Sciences Kampala. Samples collected after midday were refrigerated till the next day.

Thus, the study analysis included study participants with both EM and spot sputum samples submitted for culture. 

#### 2.1.1. Laboratory Procedures

Sputum samples were processed according to standard procedures[15] and simultaneously inoculated in Lowenstein Jensen (LJ) (Becton and Dickson, Franklin Lakes, NJ, USA) and Mycobacterium Growth Indicator Tube (MGIT) (Becton and Dickson, Franklin Lakes, NJ, USA) culture bottles with growth media as described elsewhere [15, 16]. Smears for fluorescent microscopy were also done according to standard procedures [15]. 

For purity, MGIT-positive cultures were further sub-cultured at 37°C on blood agar for 24 hours and Ziehl Neelsen (ZN) smears done. Cultures which were ZN-positive and pure (i.e., no growth on blood agar) were subjected to Capilia TB Neo (TAUN, Numazu, Japan) for identification of the MTBC [17]. Cultures with growth on blood agar but also ZN-positive were sub-cultured again as described above; the persistent ZN-negative cultures with growth on blood agar were considered contaminated. All ZN-positive, blood agar negative, and Capilia TB Neo negative samples were regarded Mycobacteria Other Than Tuberculosis (MOTT); these were subjected to the Genotype AS assay (Hain Lifescience, Nehren, Germany) for speciation. Otherwise absence of colonies on LJ or fluorescence in MGIT was considered negative. The data were entered into a computerized laboratory access database linked with patient records on sample reporting form.

#### 2.1.2. Data Analysis

The primary outcome of our analysis was culture positive TB, defined as MTBC positive upon LJ or MGIT culture from either spot or EM samples. Laboratory data were analyzed for the diagnostic yield; this referred to the number of TB cases detected by each sputum sample type (spot or EM) irrespective of whether the comparator was positive. The data were analyzed for the Incremental diagnostic yield; this referred to the yield of culture positive EM samples in terms of TB cases detected when the spot samples from the same case was negative. Contamination rate was calculated as the total number of cultures per sample contaminated over the total number of cultures per sample type inoculated.

Statistical analysis was performed with Stata SE software version 11 (Stata Corp LP, College station TX, USA). The unit of analysis was the sample type to show the diagnostic yield from each and Incremental yield for EM sputum samples collected for culture. A *P*-value of <0.05 was considered statistically significant.

#### 2.1.3. Ethical Considerations

This study operated under a waiver of consent since it involved secondary data analysis from the major study that previously obtained ethical approval from the Makerere University College of Health Sciences School of Public Heath Institutional Review Board (IRB) and the Uganda National Council for Science and Technology (UNCST). 

## 3. Results

**Figure 1:**
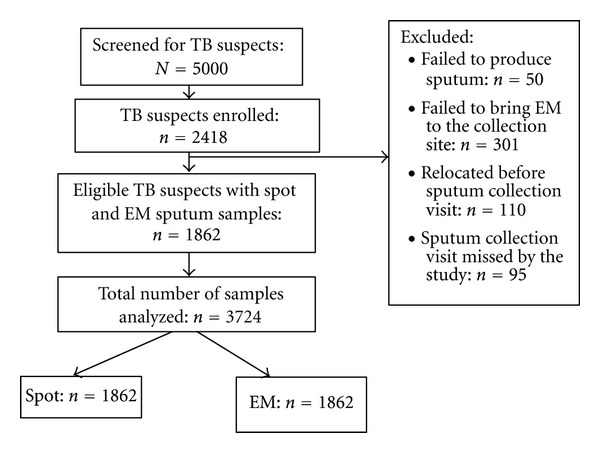
Study flow chart for 1862 adolescent TB suspects based on spot and EM sputum samples.

A total of 5000 adolescent participants were screened for TB, of whom 2418 (48.4%) met the study criteria for a TB suspect. Of the 2418 TB suspects, 803 (33.2%) had a positive TST, 237 (57.0%) had contact with a TB patient, and 1378 (57.0%) had at least one TB symptom. Furthermore, 556 (22.9%) were excluded due to challenges with sample collection; 50 (2.1%) had dry cough hence did not provide sputum, 301 (12.4%) failed to deliver an EM sample, 110 (4.5%) relocated prior to sputum collection, while 95 (3.9%) missed the study visit to the collection point. Thus, the analysis was limited to 1862 eligible participants who had both the EM and spot samples, Figure 1. 

Of the 1862 participants, six (0.3%) were MTBC-positive based on smear microscopy while 21 (1.1%) were MTBC-positive based on culture (confirmed by Capilia Neo TB assay as described in methods) hence they were regarded as true TB cases (Table 1).

### 3.1. Mycobacterial Yield of Spot and Early Morning Sputum Samples

Based on LJ culture, only six (0.4%) samples were MTBC-positive meaning the TB case detection based on LJ-positive culture were six cases: these were also positive by smear microscopy. Of the six LJ-positive cases, one (0.1%) was from the spot sample, two (0.2%) from EM, (*P* < 0.001) while three (0.5%) were from both spot and EM samples. The LJ contamination rate for spot samples was 1.6% while it was 2.9 % for the EM samples (*P* = 0.008). 

On the other hand, MGIT culture detected 21 TB cases (including the six LJ-positive ones); six (0.3%) on spot, EM 12 (0.6%), and three (0.2%) on both spot and EM samples (*P* < 0.001). Furthermore, with MGIT, 225 MOTT (12.1%) were also detected of which 106 (5.7%) and 119 (6.4%) (*P* < 0.001) were from spot and EM samples, respectively, (Table 2). 

The MOTT were speciated as follows: *M. fortuitum* 143 (63.6%); *M. Szugai* 32 (14.2%); *M. gordonae *21 (9.3%); *M. intracellulare* 11 (4.9%); *M. scrofulaceum* 7 (3.1%); *M. lentiflavum* 7 (3.1%); *M. peregrinum* 4 (1.8%).

Overall, 436/1862 (23.4%) of the spot and 499/1862 (26.8%) (*P* < 0.001) of the EM sample cultures were contaminated (Table 2). The most common contaminants were Gram positive cocci. 

### 3.2. Diagnostic Yield of TB for the Spot and the Incremental Yield of the Early Morning Sputum Sample Cultures

The TB diagnostic yield of the spot sample was 4 (19.0%) and 12 (57.1%) on LJ and MGIT, respectively. The incremental diagnostic yield (not total) of the EM sample was 2 (9.5%) and 12 (42.9%) (*P* < 0.001) on LJ and MGIT, respectively (Table 3).

## 4. Discussion

This study has revealed that among adolescent TB suspects in rural Uganda, an EM sputum sample culture has a higher incremental diagnostic yield. However, the study also found that EM culture also detects more MOTT in comparison with the spot sample. The diagnostic gain of the EM sputum culture given a negative spot sputum sample culture was 9.5% on LJ culture and 42.9% on MGIT.

Given the sensitivity of sputum culture especially with liquid systems (MGIT) over smear microscopy, one would imagine the incremental diagnostic yield of the EM sputum sample to be negligible; however, this is contrary to our findings which are in agreement with the previous studies by either microscopy or culture [7, 18–20]. Findings in this study further agree with previous studies which reported a significant gain from the EM sample [21–23]. This may be due in part to the accumulation of sputum in the lungs overnight, resulting in a concentration of bacilli in the EM samples. In contrast, patients may be more active during the day and may shed bacilli intermittently, thus reducing the yield of bacilli in spot sputum samples. These facts further support guidelines to examine EM samples in addition to spot sputum samples before declaring a TB suspect as negative for pulmonary TB.

Solid culture detected less TB cases and it was less contaminated compared to MGIT and this difference, in terms of sample type, was in agreement with previous studies [21, 24, 25]. However, Tortoli et al. documented the opposite with LJ culture [26]. The low yield of LJ in comparison with MGIT is unlikely to be attributed to decontamination procedures as samples were processed similarly using the same procedures and with the same homogenized sputum samples inoculated in both culture media. The lower contamination rate could be due to the fact that we used commercially prepared LJ which was properly quality-controlled and was less likely to have attracted contaminants.

 The high contamination rate with MGIT could partly be due to the fact that this method uses highly nutritious medium that easily supports growth of other bacteria. Alternatively, the long distance from the sputum sample collection site (3 hrs drive) to the laboratory could have contributed in that it facilitated rapid multiplication of normal flora, which are mainly Gram positive cocci [26]. We observed less contamination with spot samples (which were supervised at collection sites) when compared with the EM samples (which were not unsupervised at collection sites); however, this may not be significant since we did not provide patients with water or other materials to minimize normal flora prior to expectoration. Nevertheless, this finding is supported by similar findings in a previous study [27]. It is also worth noting that high contamination rates may have contributed to underestimation of TB cases by MGIT and increased the cost of doing the test. Furthermore, the increased yield of the second sputum culture could be partly due to the EM samples detecting those which were contaminated on spot; however contamination is unlikely to explain the increased yield of EM culture since a similar scenario of increased yield was also documented by previous studies with low contamination rates [21, 22].

In agreement with previous studies [28], we found that LJ may have an unfavorable environment to support MOTT growth since all the isolated mycobacteria were MTBC [28] and a significant number of MOTT were isolated with MGIT. This is mainly due to the fact that MGIT is more sensitive and facilitates rapid multiplication of fast growing MOTT than LJ. That is why WHO emphasizes rapid identification of any MGIT which is ZN positive to be identified before reporting the results [29]. However this is contrary with another study which documented almost similar number of MOTT isolated on LJ and MGIT cultures [26]. This may be attributed to regional differences in distribution of MOTT as most MOTT species from that study are different from those identified in ours.

Since most mycobacteria are similar phenotypically, the prevalence of MOTT in this study population is worrying in that they may distort the immunogenicity data in the planned TB vaccine trials. Furthermore, much as we did not document any disease due to MOTT infection, studies on MOTT are needed since diseases due to these organisms have been documented in other settings [30]. Additionally, as the number of participants who failed to return with an EM sample in this study was high 301/2418 (12.4%), future studies considering other sputum sample collection strategies, such as, the “Front Loading” strategy would also be important.

Our study had some limitations; first, we did not separate mycobacterial yield of spot and EM sputum samples collected at baseline and follow-up; therefore, differences in sample composition may have occurred at different time points during the study. Furthermore, the main study documented few laboratory confirmed TB cases. This may be different when other forms of TB diagnosis including clinical diagnosis are considered. However, given a bigger sample size, our findings provide significant information for future studies on TB among adolescents.

The samples analyzed were collected in a research setting with supervision of the spot sample collection as well as being analyzed in a purely research BSL-3 TB culture facility; hence, much as our findings have implications for controlled research settings like clinical trials, they may not represent what happens in routine practice.

Additionally, we identified MTBC with Capilia TB Neo, which may give false negatives as documented by previous studies [30, 31]; however, all MOTT and MTBC were re-confirmed using the Line Probe Hain CM and AS genotype assays.

In conclusion, early morning sputum culture has a high incremental diagnostic yield for TB among adolescents in Uganda. Among TB suspected adolescents, early morning sputum in addition to spot sample culture is necessary for better TB case detection. MGIT system cultivated all the MTBC cases as well as MOTT and majority were from early morning sputum samples. 

## Figures and Tables

**Table 1:** Characteristics of the six patients with MTBC-positive smears and the 21 patients with MTBC-positive culture sputum sample type among adolescent TB suspects in rural Uganda (*n* = 1862).

Characteristics	Smear positive*		Culture positive (MTBC)*		
	Spot *n* (%) of Total	EM *n* (%) of Total	Spot *n* (%) of Total	EM *n* (%) of Total	Total enrolled *n* (%) of Total
Total	3	6	9	15	1862
Sex					
Male	1 (33.3)	4 (66.7)	4 (44.4)	7 (46.7)	1069 (57.4)
Female	2 (66.7)	2 (33.3)	5 (55.6)	8 (53.3)	793 (42.6)
Age ranges					
12–14	0 (0.0)	1 (16.7)	5 (55.6)	1 (6.7)	805 (43.2)
15-16	3 (100)	4 (66.7)	4 (44.4)	8 (53.3)	543 (29.1)
17-18	0 (0.0)	1 (16.7)	0 (0.0)	7 (46.7)	514 (27.6)
Volume					
≤5 mL	1 (33.3)	3 (50.0)	3 (33.3)	9 (60.0)	1226 (65.8)
>5 mL	2 (66.7)	3 (50.0)	6 (66.7)	6 (40.0)	636 (34.2)
Consistency					
Salivary	2 (66.7)	3 (50.0)	4 (44.4)	7 (46.7)	872 (46.8)
Mucosalivary	1 (33.3)	3 (50.0)	3 (33.3)	2 (13.3)	350 (18.8)
Mucoid	0 (0.0)	0 (0.0)	1 (11.1)	2 (13.3)	360 (19.3)
Mucopurulent	0 (0.0)	0 (0.0)	1 (11.1)	3 (20.0)	213 (11.4)
Purulent	0 (0.0)	0 (0.0)	0 (0.0)	1 (6.7)	67 (3.6)

EM: Early morning, MTBC: mycobacterium tuberculosis complex, *n*: number, %: percentage; *The 3 smear positive on spot were also detected by the early morning sample, also 3 participants were positive on both spot and early morning sputum cultures.

**Table 2:** Mycobacterial yield from EM and spot sputum samples by different culture methods (*n* = 1862).

Culture method	Results	Spot samples *n* (%)	EM samples *n* (%)	EM and spot samples *n* (%)	Total *n* (%)
LJ (*P* < 0.001)	MTBC positive	1 (0.1)	2 (0.1)	3 (0.2)	6 (0.3)
	MOTT	0 (0.0)	0 (0.0)	0 (0.0)	0 (0.0)
	Culture negative	1831 (98.3)	1806 (97.0)	1859 (99.8)	1772 (95.2)
	Contaminated	30 (1.61)	54 (2.9)	0 (0.0)	84 (4.5)

MGIT (*P* < 0.001)	MTBC positive	6 (0.3)	12 (0.6)	3 (0.2)	21 (1.1)
	MOTT	106 (5.7)	119 (6.4)	0 (0.0)	225 (12.1)
	Culture negative	380 (20.4)	301 (16.2)	1859 (99.8)	681 (36.6)
	Contaminated	436 (23.4)	499 (26.8)	0 (0.0)	935 (50.2)

EM: early morning; MTBC: mycobacterium tuberculosis complex, MOTT: mycobacterium other than tuberculosis, LJ: Lowenstein Jensen, MGIT: mycobacterium growth indicator tube. IY: incremental yield, LJ: Lowenstein Jensen, MGIT: mycobacterium growth indicator tube.

**Table 3:** Diagnostic yield of TB for the spot and incremental diagnostic yield (not total) of EM sputum sample cultures (*n* = 21).

Culture method	Yield of spot *n* (%)	Incremental diagnostic yield for EM (observed) *n* (%)	Total number of TB cases detected *n* (%)
LJ	4 (19.0)	2 (9.5)	6 (28.6)
MGIT	12 (57.1)	9 (42.9)	21 (100)

*n*: number, IY: incremental yield, LJ: Lowenstein Jensen, MGIT: mycobacterium growth indicator tube, EM: early morning.

## References

[1] World Health Organization Global tuberculosis control report, 2011. http://www.who.int/tb/publications/global_report/2011/gtbr11_full.pdf.

[2] Didilescu C, Ibraim E, Tigau M (1997). The epidemiological profile and current evolutionary trends in tuberculosis in adolescents (15–19 years old) in the capital. *Pneumoftiziologia*.

[3] Nemir RL (1986). Perspectives in adolescent tuberculosis: three decades of experience. *Pediatrics*.

[4] Kam A, Ford-Jones L, Malloy P, Khan K, Kitai I (2007). Active tuberculosis among adolescents in Toronto, Canada: clinical features and delays in diagnosis. *Pediatric Infectious Disease Journal*.

[5] De Pontual L, Balu L, Ovetchkine P (2006). Tuberculosis in adolescents: a French retrospective study of 52 cases. *Pediatric Infectious Disease Journal*.

[6] Byeon YLJ, Chul LJ, Young Y (2007). Three cases of pulmonary and/or intestinal tuberculosis in adolescents. *Korean Journal of Pediatric*.

[7] http://www.who.int/tb/publications/2006/istc_report.pdf.

[8] Harries AD, Mphasa NB, Mundy C, Banerjee A, Kwanjana JH, Salaniponi FML (2000). Screening tuberculosis suspects using two sputum smears. *International Journal of Tuberculosis and Lung Disease*.

[9] Getahun H, Harrington M, O’Brien R, Nunn P (2007). Diagnosis of smear-negative pulmonary tuberculosis in people with HIV infection or AIDS in resource-constrained settings: informing urgent policy changes. *The Lancet*.

[10] Dowdy DW, Chaisson RE, Maartens G, Corbett EL, Dorman SE (2008). Impact of enhanced tuberculosis diagnosis in South Africa: a mathematical model of expanded culture and drug susceptibility testing. *Proceedings of the National Academy of Sciences of the United States of America*.

[11] Apers L, Mutsvangwa J, Magwenzi J (2003). A comparison of direct microscopy, the concentration method and the Mycobacteria Growth Indicator Tube for the examination of sputum for acid-fast bacilli. *International Journal of Tuberculosis and Lung Disease*.

[12] http://www.who.int/tb/publications/2006/tbhiv_recommendations.pdf.

[13] Woods GL (2002). The mycobacteriology laboratory and new diagnostic techniques. *Infectious Disease Clinics of North America*.

[14] Mandalakas AM, Starke JR (2005). Current concepts of childhood tuberculosis. *Seminars in Pediatric Infectious Diseases*.

[15] Strong BE, Kubica GP Isolation and identification of *Mycobacterium tuberculosis*: a guide for the level II laboratory. http://books.google.co.ug/books?id=_VurMQEACAAJ.

[16] Siddiqi SH http://www.finddiagnostics.org/export/sites/default/resource-centre/find_documentation/pdfs/mgit_manual_nov_2007.pdf.

[17] Hirano K, Aono A, Takahashi M, Abe C (2004). Mutation including IS6*110* insertion in the gene encoding the MPB64 protein of Capilia TB− negative *Mycobacterium tuberculosis* isolates. *Journal of Clinical Microbiology*.

[18] Strategic and Technical Advisory Group for Tuberculosis WHO Report on conclusions and recommendations. http://www.who.int/tb/events/stag_report_2007.pdf.

[19] Rohit S, Mukerjee S, Neeta S, Sharma PP (2001). Diagnosis of tuberculosis under RNTCP: examination of two or three sputum specimens. *Indian Journal of Tuberculosis*.

[20] Schoch OD, Rieder P, Tueller C (2007). Diagnostic yield of sputum, induced sputum, and bronchoscopy after radiologic tuberculosis screening. *American Journal Respiratory Critical Care Medicine*.

[21] Monkongdee P, McCarthy KD, Cain KP (2009). Yield of acid-fast smear and mycobacterial culture for tuberculosis diagnosis in people with human immunodeficiency virus. *American Journal of Respiratory and Critical Care Medicine*.

[22] Moore DAJ, Evans CAW, Gilman RH (2006). Microscopic-observation drug-susceptibility assay for the diagnosis of TB. *The New England Journal of Medicine*.

[23] Ssengooba W, Kiwanuka N, Kateete DP (2012). Incremental yield of serial sputum cultures for diagnosis of tuberculosis among HIV infected smear negative pulmonary TB suspects in Kampala, Uganda. *PLoS ONE*.

[24] Chihota VN, Grant AD, Fielding K (2010). Liquid vs. solid culture for tuberculosis: performance and cost in a resource-constrained setting. *International Journal of Tuberculosis and Lung Disease*.

[25] Lee JJ, Suo J, Lin CB, Wang JD, Lin TY, Tsai YC (2003). Comparative evaluation of the BACTEC MGIT 960 system with solid medium for isolation of mycobacteria. *International Journal of Tuberculosis and Lung Disease*.

[26] Tortoli E, Cichero P, Piersimoni C, Simonetti MT, Gesu G, Nista D (1999). Use of BACTEC MGIT 960 for recovery of mycobacteria from clinical specimens: multicenter study. *Journal of Clinical Microbiology*.

[27] Maciel ELN, do Prado TN, Peres RL, Palaci M, Johnson JL, Dietze R (2009). Guided sputum sample collection and culture contamination rates in the diagnosis of pulmonary TB. *Jornal Brasileiro de Pneumologia*.

[28] Worodria W, Anderson J, Cattamanchi A (2011). The role of speciation in positive Lowenstein-Jensen culture isolates from a high tuberculosis Burden country. *PLoS ONE*.

[29] World Health Organization http://www.who.int/tb/laboratory/policy_liquid_medium_for_culture_dst/en/index.html.

[30] McCarthy KD, Cain KP, Winthrop KL (2012). Nontuberculous mycobacterial disease in patients with HIV in Southeast Asia. *American Journal Respiratory Critical Care Medicine*.

[31] Muchwa C, Akol J, Etwom A (2012). Evaluation of Capilia TB assay for rapid identification of *Mycobacterium tuberculosis* complex in BACTEC MGIT 960 and BACTEC, 9120 blood cultures. *BMC Research Notes, article 44*.

